# Lipid Biomarkers in Depression: Does Antidepressant Therapy Have an Impact?

**DOI:** 10.3390/healthcare10020333

**Published:** 2022-02-09

**Authors:** Fidelis Christin Stuchtey, Andrea Block, Francis Osei, Pia-Maria Wippert

**Affiliations:** 1 Department of Human Medicine, Paracelsus Medical Private University, 5020 Salzburg, Austria; fidelis.stuchtey@stud.pmu.ac.at; 2 Medical Sociology and Psychobiology, Department of Health and Physical Activity, University of Potsdam, 14469 Potsdam, Germany; anblock@uni-potsdam.de (A.B.); francis.osei@uni-potsdam.de (F.O.); 3 Faculty of Health Sciences Brandenburg, Joint Faculty of the University of Potsdam, The Brandenburg Medical School Theodor Fontane, Brandenburg University of Technology Cottbus, Senftenberg, 14469 Potsdam, Germany

**Keywords:** major depressive disorder, antidepressants, high density lipoprotein cholesterol, HDL, low density lipoprotein cholesterol, LDL, cholesterol, triglycerides, lipids

## Abstract

Studies have revealed mixed results on how antidepressant drugs affect lipid profiles of patients with major depression disorder (MDD). Even less is known about how patients respond to a switch of antidepressant medication with respect to their metabolic profile. For this, effects of a switch in antidepressants medication on lipid markers were studied in MDD patients. 15 participants (females = 86.67%; males = 13.33%; age: 49.45 ± 7.45 years) with MDD and a prescribed switch in their antidepressant medication were recruited at a psychosomatic rehabilitation clinic. Participants were characterized (with questionnaires and blood samples) at admission to the rehabilitation clinic (baseline, T0) and followed up with a blood sample two weeks (T1) later. HDL, LDL, total cholesterol, and triglycerides were determined (T0), and their change analyzed (Wilcoxon test) at follow up (T1). Decrements in HDL (*p* = 0.041), LDL (*p* < 0.001), and total cholesterol (*p* < 0.001) were observed two weeks after a switch in antidepressant medication. Triglycerides showed no difference (*p* = 0.699). Overall, LDL, HDL, and total cholesterol are affected by a change in antidepressant drugs in patients with MDD. These observations are of clinical relevance for medical practitioners in the planning and management of treatment strategies for MDD patients.

## 1. Introduction

Major depressive disorder (MDD) is a pathological condition that is estimated to affect over 264 million people of all ages worldwide (WHO, 2020) [1]. The prevalence rate of self-reported medical diagnosed depression in Germany is estimated at 8.1% for females and 3.8% for males, respectively [2]. In Germany, the annual economic burden of depression per-patient is amounted to EUR 5047 (95% confidence interval (CI) 3214–6880) [3].

To make matters worse, there is growing evidence that major depressive disorder is an independent risk factor for cardiac death, cardiovascular events, and cardiovascular disease (CVD) [4]. Vice versa, patients with depression more often develop CVD and are prone to premature mortality [5,6]. Additionally, alterations in lipid homeostasis, such as increased low-density lipoprotein cholesterol (LDL) and raised triglycerides, were found to act as an independent risk factor for incident and recurrent coronary heart disease (CAD) [4]. There are some mixed results from epidemiologic and intervention studies suggesting that low levels of cholesterol due to lipid lowering drugs are associated with depressive symptoms [7]. Furthermore, hypocholersterolemia was found to be associated with a higher risk of MDD and risk of death from suicide [8,9]. Growing evidence suggests an inter-relationship between lipid homeostasis and the pathoetiology of major depression, and it is hypothesized that alterations of the lipid homeostasis, especially in total cholesterol levels, have an impact on the serotonin neurotransmission in the brain [10].

**Figure 1:**
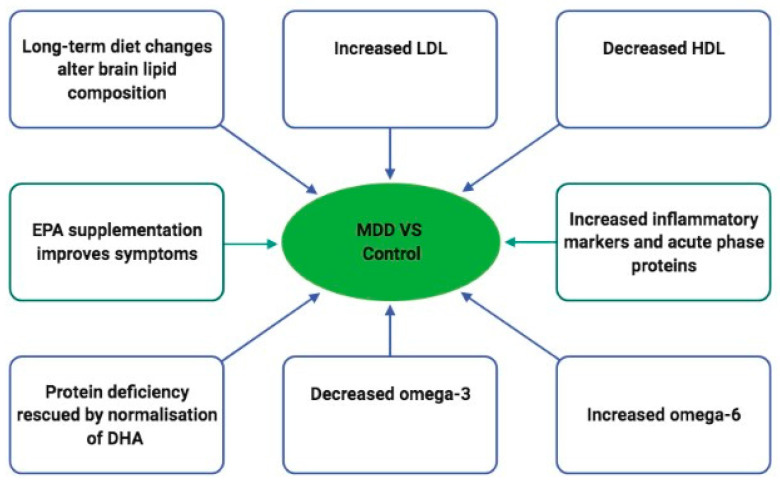
The figure above indicates lipid biomarkers that were found to differ between patients with depressions versus control subjects without depression. Patients with MDD show increased LDL and decreased HDL, increased inflammatory markers and omega 6 fatty acids, where decreased omega 3 fatty acids are in opposition to the healthy control group. Adapted from [12].

Depressive disorders themselves appears to change the lipid markers [11]. However, there is an indication that a clear distinctive profile of circulating lipids is connected to depression with antidepressant drugs [11]. Parekh et al. suggested that there is a potential scope for the use of peripheral biomarkers, such as HDL, LDL, cholesterol, and triglycerides, in the diagnosis, stratification, and treatment of MDD [12] (see Figure 1). This indicate a relationship between metabolic imbalances and depression, but the kind of relation still needs to be evaluated. Whilst HDL increases due to anti-depressants usage, LDL, which delivers cholesterol into the periphery, decreases after SSRI use [13]. The therapy of MDD is based on three building blocks: (1) pharmacotherapy, (2) psychotherapy, and (3) sociotherapy in chronic courses of disease [14]. Although the German National S3 Guideline for the treatment of MDD recommends antidepressants medication for the treatment of severe episodes (ICD-10 code: F32.2, F32.3), there has been a quintupling in prescriptions since 1995 [15,16,17]. Treatment guidelines by the American Psychiatric Association and Agency for Health Care Policy and Research recommend a treatment with antidepressants for at least four to nine months after depressive symptoms resolve to prevent relapse [18,19]. Antidepressant drugs are an effective treatment, but up to 75% of its treatment effect are attributable to unspecific and placebo effects (especially in mild to moderate depressions; ICD-10 code: F32.0, F32.1). Almost all approved antidepressants in Germany effectuate a change in serotonin and noradrenalin concentrations in the synaptic cleft through different pathways and mechanisms, but it is still unknown how this leads to the temporally delayed antidepressant effect. Therefore, the choice of the antidepressant drug is often determined considering its side effect profile [14].

A substantial body of evidence implies that weight gain is at least partly related to the blocking effects of antipsychotic medication on serotonin- and histamine-mediated neurotransmission [20,21]. There has been evidence of decreased HDL, cholesterol, triglycerides, and hyperglycemia and increased chronicity of depression in antidepressant users [21]. Although it is established that some antidepressants induce well-documented side effects such as weight gain and alteration in the glucose-insulin homeostasis, the effects of depression and antidepressant medication on lipid metabolism and homeostasis are poorly understood [7]. The most often prescribed antidepressants are monoamine-reuptake-inhibitors (MRI) of which selective serotonin re-uptake inhibiter (SSRI) are the first choice as they have lesser side effects than their tricyclic drugs (TCAs) counterpart—especially regarding weight gain [22,23]. There is evidence that tricyclic antidepressants have an impact on cholesterol levels, but the results are contradictory. However, it is recommended by the guidelines [24] that after reaching the standard dosage a first assessment of antidepressants time delayed efficacy is reliable three to four weeks (6 weeks for older patients) later [25]. Interestingly, a meta-analysis revealed that an early improvement of depressive symptoms with antidepressant medication predicted a stable response and remission in MDD therapy [26]. Thus, understanding the effect of antidepressant drugs on clinical symptoms of depressions and other body systems such as metabolic, immunoregulatory, and humoral are of vital importance [27,28,29].

All these findings indicate that metabolic markers may be useful in understanding depression and treatment response to antidepressants better, given that weight gain markedly increases the probability of developing depression [30,31]. Evidence suggests that diets high in saturated fat and relatively low in polyunsaturated and monounsaturated fatty acids contribute to the pathogenesis of both mood and metabolic disorders by weight gain, which can be seen in the lipid panel (HDL, LDL, cholesterol, and triglycerides) [31]. In the wake of those findings, this study aims to investigate how a prescribed change in antidepressant medication affects lipid homeostasis in a short-term period in depressed adults. The work aspires to broaden the understanding of the association between antidepressants use and lipid markers, which may be of clinical relevance for practitioners.

## 2. Materials and Methods

### 2.1. Participants

A sub-sample of *n* = 15 patients (females = 86.67% and males = 13.33%) from a psychosomatic rehabilitation clinic within the Depreha study (*n* = 206 for further details see: [32]) was recruited and analyzed. Inclusion criteria of the Depreha study were age (18–65 years), the diagnosis of depressive episodes (ICD-10 F32.x or F33), dysthymia (F34.1) or adjustment disorder with depressed mood (F43.21) and a history of more than 21 days on sick leave due to the diagnosed diseases. Exclusion criteria were: pregnancy, patients with hormonal therapy (excluding the oral contraceptives or thyroid medication), or patients with other significant primary diagnosis such as mental retardation, metabolic disorders, dementia, neurologic disorders, drug abuses, psychotic disorders, and personality disorders. In this analysis, only patients with diagnosed MDD and a prescribed change of the antidepressant medication at admission to the clinic were recruited. Participants (age: 49.45 ± 7.45) were measured at baseline (admission to the clinic, T0) and followed up two weeks (T1) later. All participants were fully informed in written and verbal form about the intent and content of the study. All participants gave written informed consent.

### 2.2. Study Design

The study objectives presented here were investigated at baseline (T0, questionnaires and blood samples) and after 2 weeks (T1, blood samples). The study design is described in more detail elsewhere [32]. All clinical investigations were conducted according to the principles expressed in the Declaration of Helsinki, 1964. Final ethical approval was provided by the major institutional ethics review board of the University of Potsdam, Germany (number 13/15).

### 2.3. Psychosocial Measurement

Questionnaires were used to assess the sociodemographic characteristics and antidepressant medication of participants. The Beck Depression Inventory-II (BDI-II) was used to assess depressive symptoms and severity [33]. The BDI-II has been validated in Germans clinicals and non-clinical samples with internal consistency of (Cronbach’s alpha ≥ 0.84), re-test reliability exceeding r ≥ 0.75 [34].

### 2.4. Anthropometric Measurement

The height of subjects was measured to the nearest 0.5 cm using a stadiometer and the weight measured to the nearest 0.1 kg on a medical scale (Seca, Hamburg, Germany).

### 2.5. Blood Sampling

Blood samples (T0, T1) were drawn in the morning hours (7–9 am) from the antecubital vein and collected in plain blood collection tubes or tubes containing EDTA for sub sequent analysis by laboratory partners. Lipid profiles (i.e., HDL, LDL, cholesterol, and triglycerides) was assessed using Roche/Hitachi Cobas 701/702 (F. Hoffmann-La Roche, Ltd., Basel, Switzerland) via enzymatic colorimetric assays.

### 2.6. Statistical Analysis

All data were analyzed using (SPSS v. 25.0, Chicago, IL, USA). Descriptive statistics are presented in means (M) and standard deviation (SD). Wilcoxon test was used to compare the lipid markers between baseline (T0) and after two weeks (T1). The level of significance was set at *p* < 0.05.

## 3. Results

**Figure 2:**
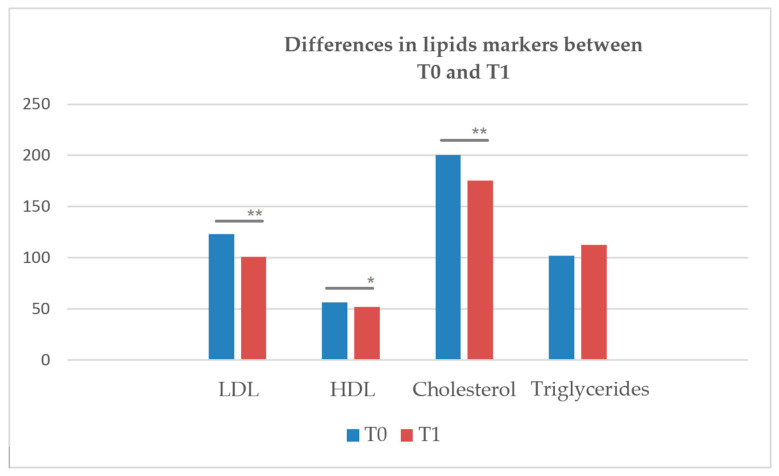
Graphical presentation between lipid markers in baseline (T0) vs. 2 weeks (T1). * Significant at *p* = 0.05, ** Significant at *p* = 0.01.

Descriptive statistics of the sample and sociodemographic characteristics are shown in Table 1 and Table 2, respectively. The changes of the lipids over time between baseline (T0) and after two weeks (T1) are shown in Table 2 and Figure 2. A decrement between baseline levels (T0) and two weeks follow-up (T1) after change in antidepressant medication were observed for HDL (*p* < 0.035), LDL (*p* < 0.001), and cholesterol (*p* < 0.001). Triglycerides show no difference (*p* < 0.363) between the two time points. Weight (T0: 80.58 kg (SD 24.6); T1: 80.15 kg (SD 23.24)) and BMI (T0: 27.83 (SD 7.06); T1: 27.80 (SD 6.57)) were unchanged between baseline (T0) and follow-up (T1).

## 4. Discussion

The aim of the present study was to understand the short-term effect of a change in antidepressant medication on lipid markers in depressed adults as this is of relevance for practitioners, especially in MDD patients with comorbidities such as cardiovascular diseases or Morbus Addison. Two weeks after a prescribed change in antidepressant medications, a decrement in HDL, LDL, and cholesterol (but not in triglycerides) was found.

This alteration in lipid homeostasis may indicate a short-term effect of a change or adjustment of antidepressant medication. There is a limited number of studies that examined the short-term effects (four to six weeks) of antidepressant medication on lipid profile in depressive patients [35]. To the best of our knowledge, there is only one other study that examined short effects of an antidepressant medication on lipid profile continuously for seven days in a laboratory setting and found decrements in cholesterol, HDL, LDL, and no effect on triglycerides [36]. Although the study by Hennings et al. [35] controlled for diet (standardized), sleep–wake cycle, and exercise, we found similar effects on lipid profiles after a change in antidepressant medication in a naturalistic, clinical setting. These results are in line with increased levels of HDL [37] and LDL [7] four or five weeks after antidepressant onset, respectively. Additionally, evidence shows an association between the reduction in cholesterol levels in MDD patients taking escitalopram for six weeks [38]. Although we observed significant decrease in HDL levels after the two weeks, other studies found no changes in HDL levels after antidepressant treatment (sertraline, escitalopram, fluoxetine, and venlafaxine) after 8 weeks [39], whereas 35 days administration of amitriptyline or paroxetine did not alter cholesterol [7]. This could be due to the different duration of antidepressant administration and lipid profile measurements of the studies compared to the two weeks intervention employed in our study.

In a review, McIntryre et al. [4] summarized the studied effects of different types of antidepressants on lipid homeostasis: SSRI were found to decrease levels of total cholesterol and triglycerides compared to controls; mixed results were discovered concerning TCA effects on lipid profiles. Mirtazapine, a tetracyclic antidepressant, showed no effect on lipid profiles in depressed patients compared to a healthy control group [40].

Although there is still a lack of well-designed, randomized, controlled, and prospective studies that evaluate the effects of antidepressants (TCA, SSRI, SNRI, MAOI, and atypical antidepressants) on lipid homeostasis in different populations, it was stated that antidepressant alter lipid profiles in a clinically relevant way, but the effects were mostly attributable to the medication-induced alteration in body weight [4]. On the contrary, a more recent, highly standardized, and controlled study revealed a weight-gain-independent metabolic effect of mirtazapine on lipid and glucose profiles in healthy men [35]. Our study confirms these more recent results in depressive patients in a naturalistic, clinical setting.

Although the sample under study is small, these findings are clinically relevant as dyslipidemia is another risk factor that contributes to mortality and morbidity in psychiatric patients [41]. An interesting finding from the study is the reduction in LDL and cholesterol levels. Lower levels of HDL have been linked to a higher risk for CVD mortality and morbidity [42,43,44]. Thus, the findings of the study may suggest antidepressant use reduces HDL levels, which may lead to the risk of CVD diseases.

Unlike most other studies on the effect of antidepressants on lipid profiles, our study examined a prescribed switch in antidepressant medication patients with diagnosed depression, and not antidepressant-naïve populations. It should be noted that a switch in antidepressant medication is often inevitable and medically indicated due to lack of improvement in depressive symptoms or side-effect burden. Therefore, our study in a more naturalistic setting is closer to the daily routines and challenges of MDD treatment in clinical settings. However, the potential impact of antidepressant medication on lipid profiles should be taken into consideration when prescribing or switching antidepressant medication, especially for individuals with Addison’s disease and CVD whose alteration of lipids markers can cause severe health hazards. It is noteworthy that alteration of lipid profiles by antidepressant drugs may lead to decreased life expectancy, deriving from increased risk of suicide [45]. Several studies reported a link between low cholesterol levels and a higher risk of MDD, correlated with higher risk of suicide [8,46].

The pathophysiological pathways induced in depression arising from chronic hypothalamic-pituitary-adrenal axis (HPA) and inflammatory activity, leading to lipolysis, release of fatty acids, hypertriglyceridemia, and reduction in HDL, may be due to lipid dysregulations [47]. This could cause strain on the sympathoadrenal system and HPA axis, leading to a rise in circulating catecholamines and serum cortisol [47,48]. Such a rise in circulating catecholamines and serum cortisol may increase heart rate and blood pressure, leading to CVD development [6]. Additionally, cytokine profiles may be altered by antidepressant medication, leading to an alteration in lipid metabolism in MDD patients [49,50]. Such alterations in lipid metabolism by antidepressant medication has been linked to atherosclerosis pathology [51,52]. However, in every medical prescription, options are weighed for efficacy and acceptability as well as potential risks [53].

There are still open questions. Whilst our study only focused on typical depression (i.e., MDD), which may be connected to loss of appetite and insomnia, there are other subtypes of depression, such as atypical depression, that presents itself with hyperphagia and hypersomnia, and may be affected differently regarding lipid homeostasis [54]. Thus, further studies are warranted to reveal the effects of different antidepressants on lipid markers in different subtypes of depression.

Lastly, due to the inflammation associated with MDD, diet therapy is recommended to foster improvements of the lipid markers in these patients [12]. Whole grain foods rich in phytochemicals have been reported to reduce the oxidative stress which contribute to the inflammation feature of depression pathology [55]. However, as such dietary management was not offered during the rehabilitation period in our study, the results cannot be attributed to a special diet.

It should be considered that individuals with MDD are affected differently with antidepressant drugs. In general, tricyclic drugs have been reported to have higher inflammatory and metabolic alterations than SSRI drugs in a study using over 1000 MDD patients [47]. These differences may be likely due to the anti-histaminergic and adrenergic effects of tricyclic drugs leading to weight gain and possibly hypertension and dyslipidemia [56,57]. However, SSRI drugs may also increase circulating norepinephrine levels leading to an increase in heart rate [56]. Interestingly, there has been evidence of the existence of coronary heart disease and myocardial infarction in MDD patients [58].

As well as body weight, diet, and energy expenditure, further regulatory pathways may be involved in the interplay between antidepressants drugs and lipid homeostasis in depression. The neuromodulatory characteristics of pro-inflammatory cytokines were found to be a key factor in the mediation of MDD symptoms on the behavioral, neuroendocrine, and neurochemical level, leading to the “cytokine hypothesis of depression” [59]. Pro-inflammatory cytokines such as IL-1β, IL-6, and TNF-α closely interact with immune and metabolism regulatory systems and modulate the risk for diabetes mellitus type II and obesity.

They promote lipolysis, inhibiting lipid synthesis, and decrease blood lipids [59,60]. This is of vital importance in understanding pathologies such as atherosclerosis, which has been linked to lipid metabolism [60]. Thus, understanding how anti-depressant usage alters lipid markers is of clinical importance in preventing CVD. This is fruitful in people with comorbidities, such as congenital heart disease (CHD), who are prone to a 30% risk of depression and who are mostly medicated. Additionally, individuals with Addison’s disease have a high risk for depression and metabolic abnormalities both in the short and long term [11,29].

### Limitations

The results of this study must be considered in the light of some limitations. First, a switch in antidepressant medication was prescribed by the practitioners (psychiatrist or psychologist) for only 15 patients, therefore limiting those available for this specific sub-study. Although the analysis is based on a quite small sample in an uncontrolled, naturalistic setting, our results of decreasing levels in HDL, LDL, and cholesterol, and unaffected levels of triglycerides are in line with studies which rely on more participants, control groups, laboratory settings, and a longer follow-up duration.

A second limitation was the lack of information concerning antidepressant medication. Self-reports on type of antidepressant drug the patients used before admission to the rehabilitation clinic, the duration of antidepressant medication, and new type of antidepressant drug after the switch were incomplete and not verifiable by medical records. This should be considered especially with respect to the literature about different antidepressants having different effects on lipid markers [17,18,19,20,21,22,23,24,25,26,27,28,29,30,31,32,33,34,35,36,37,38,39,40].

Third, our analysis lacks a control group of depressive patients that have no switch in their antidepressant medication. Therefore, it cannot be ruled out that the observed effects are due to further influencing factors such as other therapeutic interventions at the rehabilitation clinic (psychotherapy, exercise, or a protected space) or time effects.

Furthermore, nutrition has essential effects on lipid profiles and homeostasis. Diet preferences of the participants were not accounted for in this study. However, the food supply in rehabilitation clinics is not subject to any specific diet management. To overcome the shortcomings of this study, examinations in larger and controlled study samples are needed with reliable information on antidepressant medication (history) and short-term as well as long-term follow-ups. However, our results comply with other research on the effects of antidepressant medication on lipid profiles in MDD, but our study limitations call for a more profound and expanded research.

## 5. Conclusions

The results of our study show that LDL, HDL, and cholesterol are affected by antidepressant use. Despite a small sample size and a lack of advanced information on antidepressant medication, our results are in line with recent research. These observations are of clinical relevance for medical practitioners in the planning and management of treatment strategies for MDD patients, as alterations in lipid profiles in patients with MDD were associated with higher risks of suicide and CVD.

## Figures and Tables

**Table 1:** Descriptive statistics and sociodemographic characteristics of the sample (*n* = 15).

Variables	M/N	(SD)/%
Age (years)	49.45	7.42
Gender		
Female	13	86.67
Male	2	13.33
Marital Status		
Married/in relationship	10	66.67
Single/divorced/widowed	5	33.33
Educational level		
<10 years	7	46.67
=10 years	2	13.33
10 years	6	40.00
Depression Severity ^1^		
None	0	0.00
Mild	1	6.67
Moderate	6	40.00
Severe	6	40.00
No information	2	13.33
Height (cm)	179.36	6.13
Weight (kg)	80.58	24.06
BMI (kg/m^2^)	27.83	7.06
Smoking		
Regularly	10	66.67
Not smoking	5	33.33
Drinking		
Regularly	9	60.00
Not drinking	6	40.00

M = mean, N = number, SD = standard deviation; ^1^ Classification in categories of depressive symptoms severity in accordance to the BDI-II manual [31].

**Table 2:** Differences in Lipid markers between T0 and T1.

Variables		T0	T1		Z	*p*-Value
	M	SD	M	SD		
LDL (mg/dL)	31.60	44.39	107.33	36.10	−3.297	<0.001 **
HDL (mg/dL)	55.58	16.00	51.84	14.58	−2.104	0.035 *
Cholesterol (mg/dL)	203.28	45.53	183.13	42.55	−3.233	<0.001 **
Triglyceride (mg/dL)	107.74	51.20	115.34	46.00	−0.910	0.363

Key: HDL = high density lipoprotein cholesterol; LDL = low density lipoprotein cholesterol, M = mean; SD = standard deviation. * Significant at *p* = 0.05, ** Significant at *p* = 0.01.

## Data Availability

The datasets generated for this study are available upon request to the corresponding author.
